# From Established Care to Precision Menopause Medicine: A Clinical-Actionability Framework for Contemporary Menopause Management

**DOI:** 10.3390/jcm15218674

**Published:** 2026-11-06

**Authors:** Cristian Ioan Iuhas, Mădălina-Manuela Marinescu, Corina Danciu, Cristina Mihaela Ormindean, Răzvan Ciortea, Andrei Malutan, Dan Mihu, Zorița Diaconeasa, Liora Colobatiu

**Affiliations:** 1 2nd Department of Obstetrics and Gynecology, “Iuliu Hatieganu” University of Medicine and Pharmacy, 400012 Cluj-Napoca, Romania; 2 Faculty of Food Science and Technology, University of Agricultural Sciences and Veterinary Medicine, 400372 Cluj-Napoca, Romania; 3 Department of Pharmacognosy, Faculty of Pharmacy, Victor Babeș University of Medicine and Pharmacy Timișoara, Eftimie Murgu Square, No. 2, 300041 Timișoara, Romania; corina.danciu@umft.ro; 4 Faculty of Pharmacy, “Iuliu Hatieganu” University of Medicine and Pharmacy, 400012 Cluj-Napoca, Romania; mihaela.colobatiu@umfcluj.ro

**Keywords:** perimenopause, menopause, menopausal hormone therapy, neurokinin receptor antagonists, fezolinetant, elinzanetant, precision medicine, estrobolome, phytoestrogens, vasomotor symptoms

## Abstract

Menopause care now spans established hormone therapy, mechanism-based non-hormonal drugs, behavioural interventions, and emerging microbiome- and biomarker-guided approaches. This state-of-the-art narrative review evaluated which dimensions of personalisation are clinically actionable and which remain hypotheses of precision medicine. A structured PubMed/MEDLINE search (January 2020–July 2026), updated to August 2026 and supplemented by landmark studies, current guidelines, and regulatory sources, was synthesized using a four-tier clinical-actionability framework (A, established; B, evidence-supported off-label; C, emerging; D, experimental). Menopausal hormone therapy remains the most effective treatment for vasomotor symptoms and prevents bone loss during use; the 2025–2026 FDA labelling revisions altered risk communication rather than the underlying evidence, and professional responses diverged. Fezolinetant and elinzanetant provide mechanism-based non-hormonal options, while SSRIs/SNRIs, gabapentin, cognitive behavioural therapy (CBT), CBT for insomnia, and clinical hypnosis remain evidence-supported. Exercise, yoga, acupuncture, soy, and equol are not guideline-recommended for reducing vasomotor symptom frequency despite benefits in other domains. Gut and genitourinary microbiome findings should not be conflated: pooled evidence identifies no consistent postmenopausal gut signature, whereas vaginal *Lactobacillus* depletion is more reproducible but does not reliably predict symptom resolution. Evidence-based personalisation therefore remains principally clinical; microbiome sequencing, equol testing, and multi-omic treatment selection require prospective test-guided validation.

## 1. Introduction

Menopause is a natural biological transition in a woman’s life, usually occurring between 45 and 55 years of age, and is defined retrospectively by 12 consecutive months of amenorrhea in the absence of another physiological or pathological cause. The preceding menopausal transition is characterized by variable ovarian function and may extend over several years. Because life expectancy substantially exceeds the average age at natural menopause for most women, many spend a considerable proportion of adult life after the final menstrual period.

By 2030, an estimated 1.2 billion women worldwide will be postmenopausal, with approximately 47 million entering the transition each year [1]. Vasomotor symptoms (VMSs), sleep disturbance, mood symptoms, genitourinary syndrome of menopause (GSM), sexual symptoms, and musculoskeletal complaints vary substantially among individuals and populations and can impair quality of life and work participation [2,3,4,5]. Recent meta-analytic evidence reinforces that symptom burden is associated with interacting demographic, behavioral, psychosocial, and health factors rather than a single biological determinant [3].

Contemporary menopause care is increasingly shaped by a tension between established guideline-supported management and rapidly expanding claims of individualized or “precision” treatment. Major professional societies and clinical guidelines provide increasingly detailed recommendations for menopausal hormone therapy, non-hormonal pharmacotherapy, genitourinary syndrome of menopause, premature ovarian insufficiency, and the management of women with relevant comorbidities. In parallel, emerging approaches based on neurokinin signalling, microbiome composition, phytoestrogen metabolism, pharmacokinetic variation, and multi-omic profiling have been proposed as potential tools for more individualized treatment selection. However, these approaches differ substantially in evidentiary maturity, regulatory status, reproducibility, and demonstrated clinical utility. Biologically plausible mechanisms and statistically significant associations do not necessarily translate into validated treatment-selection tools or improved patient outcomes. A critical distinction is therefore needed between personalisation that can already be implemented in routine clinical practice and precision-medicine strategies that remain investigational.

The therapeutic landscape has changed through reappraisal of MHT evidence, approval of neurokinin receptor antagonists, and increasing interest in lifestyle, nutritional, microbiome, and multi-omic approaches. The central question is therefore not only which treatments are available, but which dimensions of personalisation are clinically actionable now. For clinicians, treatment selection also requires balancing multiple therapeutic options with likely long-term adherence and socioeconomic barriers. A clear synthesis of established and emerging approaches can help place these choices in context. In this review, clinical personalisation refers to treatment selection based on symptom phenotype, age and timing, uterine status, reproductive needs, contraindications, comorbidities, drug interactions, previous response, access, cost, and informed preference. Precision medicine is used more narrowly for biomarker-, microbiome-, pharmacokinetic-, or multi-omic-guided treatment selection. The objective is to evaluate established and emerging strategies within an explicit clinical-actionability framework and to identify where plausible mechanisms have, and have not, translated into validated clinical decision tools. Accordingly, the purpose of this review is not to propose an alternative set of menopause recommendations, but to critically map current guideline-supported care against emerging precision-medicine strategies and to identify where evidence remains insufficient for routine clinical implementation.

## 2. Materials and Methods

### 2.1. Review Design and Search Strategy

The original structured narrative search of PubMed/MEDLINE covered literature published from 1 January 2020 to 31 July 2026. Search concepts included menopause, perimenopause, MHT, VMS, neurokinin receptor antagonists, fezolinetant, elinzanetant, GSM, estrobolome, gut and vaginal microbiomes, phytoestrogens, isoflavones, equol, CBT, clinical hypnosis, lifestyle, POI, breast cancer, and surgical menopause. For the present revision, targeted update searches through 20 August 2026 were performed in PubMed/MEDLINE and official regulator or professional-society sources to identify new guidelines, systematic reviews, phase 3 trials, safety communications, and labelling changes. Current documents from the FDA, EMA, North American Menopause Society, International Menopause Society, British Menopause Society, NICE, ESHRE, ASRM, and European Society of Endocrinology were cross-checked. Key publications were selected by prioritising current clinical guidelines and regulatory documents for practice recommendations, systematic reviews/meta-analyses for evidence synthesis, and pivotal randomized trials for treatment efficacy and safety. Landmark studies published before 2020 were identified from the reference lists of major contemporary guidelines and reviews and were retained when they established widely used staging criteria, mechanisms, or pivotal clinical evidence that remained relevant to current practice. Core search domains are provided in Appendix A.

### 2.2. Eligibility and Evidence Prioritization

English-language guidelines, systematic reviews, meta-analyses, randomized controlled trials, major prospective cohorts, and clinically relevant regulatory documents were prioritized. For therapeutic recommendations, priority was given to current clinical-practice guidelines, professional-society position statements, consensus documents, and regulatory sources; individual trials were primarily used to quantify efficacy, safety, or areas in which guideline recommendations remain incomplete. Observational, translational, in vitro, and animal studies were included when human interventional evidence was limited, but were treated as mechanistic or emerging evidence rather than as a basis for routine recommendations. Conference abstracts and company-reported results were retained only for investigational therapies lacking a full peer-reviewed publication and are explicitly identified as preliminary. When clinical evidence, guideline recommendations, and regulatory status did not align, these dimensions were reported separately: regulatory sources defined approved indications and mandatory safety requirements within a jurisdiction; guidelines informed clinical practice recommendations; and trial or observational evidence informed estimates of efficacy and risk. No single source type was assumed to override the others outside its appropriate role.

### 2.3. Synthesis and Limitations

Evidence was synthesized narratively according to intervention type, symptom domain, regulatory status, study design, and clinical applicability. Because this is not a systematic review, no formal risk-of-bias instrument, meta-analysis, GRADE certainty assessment, duplicate independent screening, or exhaustive study-count denominator was used. The search should therefore not be interpreted as an exhaustive enumeration of all eligible studies, and selective inclusion remains a potential source of review-level bias. To reduce this limitation, priority was given to current guidelines, regulatory documents, systematic reviews, meta-analyses, and pivotal randomized trials, with older studies retained only when they remained foundational. The review emphasizes the distinction between clinical actionability and biological plausibility. The actionability tiers below are descriptive and are not intended to replace formal guideline grading.

### 2.4. Clinical Actionability Framework

The actionability tier is assigned to a specific intervention (or test) for a specified indication and patient population; it should not be interpreted as a global grade for that intervention in all clinical circumstances. Tier A denotes established care supported by a current major guideline recommendation and/or regulatory approval for the stated indication, typically with randomized or long-term clinical evidence. Tier B denotes evidence-supported adjunctive or commonly off-label care with clinically meaningful human interventional evidence but less consistent regulatory or guideline status. Tier C denotes emerging human evidence that is mechanistically plausible or associative/interventional but is not validated for routine treatment selection. Tier D denotes preclinical, experimental, or currently unsupported strategies for the stated clinical use. Assignment reflected convergence of regulatory status, guideline position, study design, consistency, and clinical applicability. Where these sources diverged, the tier reflected the practical readiness of the specific use rather than a numerical evidence score, and the text or tables identify the relevant regulatory or guideline caveat. These clinical-actionability tiers describe readiness for practice; they are not substitutes for GRADE certainty ratings or formal risk-of-bias assessment. The framework is applied to the summary tables and the treatment-selection figure presented in the following sections.

## 3. Biology and Staging of the Menopausal Transition

The menopausal transition is driven principally by progressive depletion and reduced competence of the ovarian follicular pool. Follicular loss accelerates with reproductive ageing and is accompanied by mitochondrial dysfunction, altered DNA repair, oxidative stress, stromal and immune changes, and impaired granulosa-cell function [6,7]. These mechanisms are biologically interconnected, but their relative contribution in individual women remains incompletely defined.

**Anti-Müllerian hormone.** AMH is produced by granulosa cells of preantral and small antral follicles and is a useful marker of ovarian reserve. Concentrations decline with reproductive ageing, but AMH should not be used alone to diagnose perimenopause or menopause in otherwise healthy women of typical menopausal age. Ovarian reserve reflects follicle quantity rather than oocyte quality, and AMH does not capture the fluctuating endocrine changes that characterize the menopausal transition. Its value is greater in fertility assessment, research, and selected situations involving suspected premature ovarian insufficiency or iatrogenic ovarian damage [7].

**STRAW+10 staging.** The STRAW+10 criteria provide a widely used framework for reproductive ageing based primarily on menstrual-cycle characteristics, with supportive endocrine and ovarian-reserve markers where appropriate (Table 1) [8]. Fixed hormone thresholds should be interpreted cautiously because concentrations fluctuate substantially during the transition. Consistent with the 2025 European Society of Endocrinology guideline, routine biochemical testing is not necessary to diagnose or manage typical perimenopause or menopause in women older than 45 years; testing is more relevant in younger women when early menopause or POI is suspected [9].

**Figure 1:**
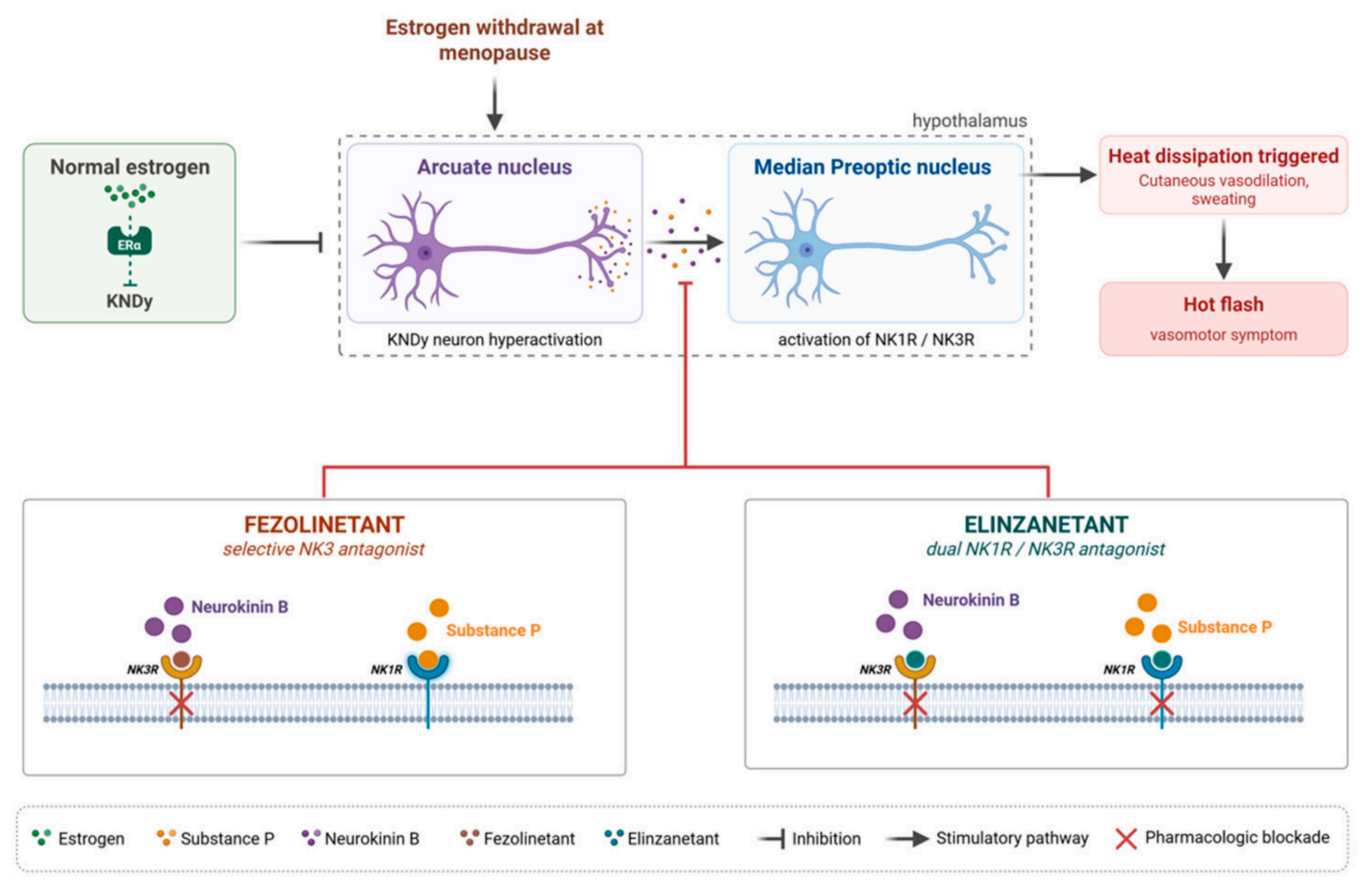
**The KNDy neuronal pathway and neurokinin-receptor antagonism in vasomotor symptoms.** Estrogen withdrawal increases activity of kisspeptin/neurokinin B/dynorphin (KNDy) neurons and enhances neurokinin B (NKB) signalling within hypothalamic thermoregulatory pathways, contributing to narrowing of the thermoneutral zone and the development of vasomotor symptoms. Fezolinetant selectively antagonises NK3R, whereas elinzanetant antagonises both NK1R and NK3R; the schematic therefore distinguishes their pharmacological targets. The relative contribution of NK1R signalling to individual vasomotor symptoms remains less completely defined than that of the NK3R pathway. NKB, neurokinin B; NK1R, neurokinin 1 receptor; NK3R, neurokinin 3 receptor.

**The KNDy neuronal circuit and vasomotor symptoms.** KNDy neurons in the hypothalamic arcuate nucleus co-express kisspeptin (KISS1), neurokinin B (NKB), and dynorphin A (PDYN), functioning as the central GnRH pulse generator. With estrogen withdrawal, KNDy neurons hypertrophy histologically, increase NKB and kisspeptin expression markedly, and hyperactivate NK3R-mediated neurotransmission, projecting to the median preoptic nucleus (MnPO) where NK3R activation triggers heat dissipation effectors: cutaneous vasodilation, sweating, and the subjective hot flash (Figure 1). This model provides the mechanistic rationale for NK3R antagonism and directly enabled development of the agents discussed in Section 5. It should nevertheless be described as the leading model rather than a complete account, because the relative contribution of NK1R-mediated signalling and of non-hypothalamic pathways to individual symptom burden remains under investigation [10,11].

**Estrogen receptors and systemic consequences.** ERα, ERβ, and GPER have overlapping but distinct tissue distributions and signaling effects. Estrogen decline contributes to accelerated bone loss, changes in body composition, adverse lipid and vascular changes, and progressive vulvovaginal and urinary symptoms [6]. Associations with cognition, neurodegeneration, sarcopenia, and cardiovascular disease are biologically plausible and epidemiologically important, but causal pathways and treatment effects differ by age, timing, formulation, and baseline health.

**Ethnic and individual variability.** The SWAN cohort and a 2026 systematic review and meta-analysis demonstrate variation in VMS presence, severity, and duration across population groups [3,12]. These differences are associated with interacting biological, psychosocial, socioeconomic, behavioral, environmental, and health-care factors. Smoking, obesity, depression, education, and income are among the factors associated with symptom patterns in pooled observational evidence [3]. Dietary patterns and microbial metabolism may contribute, but current evidence does not support attributing population differences primarily to genetics, equol-producing status, or microbiome composition.

## 4. Menopausal Hormone Therapy

MHT remains the most effective treatment for moderate-to-severe VMS and is effective for prevention of bone loss while treatment continues; low-dose vaginal estrogen is highly effective for GSM. For most healthy symptomatic women younger than 60 years or within 10 years of the final menstrual period, the benefit–risk profile is generally favourable after assessment of contraindications and individual risk factors [9,13,14,15,16]. Recent regulatory activity has changed the framing of MHT without changing the underlying evidence base, and this distinction requires explicit statement before the clinical evidence is reviewed.

**The 2025–2026 labelling change.** In November 2025, the FDA requested class-wide labelling revisions for menopausal hormone therapy and, on 12 February 2026, approved revised labels for the first six products across systemic and local categories. Risk statements on cardiovascular disease, breast cancer, and probable dementia were removed from the boxed warnings, while relevant cardiovascular and breast-cancer information remains elsewhere in systemic labels and the endometrial-cancer boxed warning remains for systemic estrogen-alone therapy in women with a uterus [17,18,19]. This was a change in risk communication rather than new randomised evidence; product-specific benefit–risk assessment by formulation, route, age, timing, comorbidity, and indication remains essential.

**International interpretation.** Regulatory and professional responses have not been identical. The Menopause Society supported removal of the boxed warning from low-dose vaginal estrogen while emphasizing individualized risk discussion for systemic therapy [20]. EMAS welcomed the FDA decision but explicitly cautioned that removal of a warning does not make MHT universally indicated [21]. The International Menopause Society supported removal for vaginal estrogen and scientific review of systemic warnings, but did not support removing all systemic warnings [22]. European clinicians should continue to follow EMA/MHRA and national product information. Across jurisdictions, MHT remains indicated principally for symptom treatment and, in selected women, prevention of bone loss rather than primary prevention of cardiovascular disease or dementia [9,13,14,15,16,23,24].

**Timing and treatment goals.** Age and time since menopause influence the absolute risks associated with systemic MHT. Initiation in younger, recently menopausal women generally has a more favorable benefit–risk profile than initiation in older women or more than 10 years after menopause. The ELITE trial showed a slower increase in carotid intima–media thickness when oral estradiol was initiated within 6 years of menopause, but not when initiated 10 or more years after menopause; this surrogate outcome does not establish MHT as a treatment for prevention of cardiovascular events [25]. Large register-based analyses of contemporary MHT show that cardiovascular risk varies by formulation and route; because these data are observational, they remain susceptible to confounding and do not establish a preventive indication [26]. MHT should be prescribed primarily for symptom treatment and, in selected women, prevention of bone loss rather than for primary prevention of cardiovascular disease or dementia.

**Route of administration and thrombotic risk.** Oral estrogen undergoes hepatic first-pass metabolism and is associated with a higher venous thromboembolism risk than non-use. Observational evidence suggests that standard-dose transdermal estradiol may have a lower thrombotic risk than oral estrogen, although direct randomized comparisons for clinical events are limited [27]. Transdermal estradiol is therefore often preferred for women with obesity, migraine, hypertriglyceridemia, gallbladder disease, or elevated baseline thrombotic risk, but route selection should remain individualized. Where systemic MHT is indicated, the formulation and route should be selected according to individual clinical factors and current product information. Transdermal 17β-estradiol avoids first-pass hepatic metabolism, but absorbed exposure can vary between preparations and individuals; this does not justify a universal preference for a single route in all patients [9,13,14,15,16,27].

**Progestogen and endometrial protection.** Women with an intact uterus receiving systemic estrogen require adequate progestogen or another evidence-based endometrial-protection strategy. Observational studies suggest that micronised progesterone and dydrogesterone may have a more favourable breast-safety profile than some synthetic progestogens, but comparative randomised evidence is limited and risk is influenced by duration and regimen [28,29]. A levonorgestrel-releasing intrauterine system can provide contraception and endometrial protection when combined with systemic estrogen, although licensing and recommended duration vary by jurisdiction. The requirement for progestogen is determined primarily by uterine and endometrial status. After total hysterectomy, estrogen-only systemic MHT is generally appropriate because endometrial protection is no longer required, although exceptions may apply in specific clinical contexts [9,13,14,15,16].

**Perimenopause-specific considerations.** MHT is not contraceptive. In women who still require contraception, options include combined hormonal contraception for medically eligible women or a progestogen method, particularly a levonorgestrel-releasing intrauterine system, combined with systemic estrogen when required for symptoms. Regimen choice should account for bleeding pattern, migraine, smoking, blood pressure, thrombotic risk, and reproductive plans.

**Unscheduled bleeding and follow-up.** Bleeding is common during the first months of continuous combined MHT and may also occur with sequential regimens. Persistent, heavy, new-onset, or recurrent bleeding after an established amenorrhoeic interval requires assessment according to age, regimen, endometrial risk factors, and local guidance. Dose, adherence, drug interactions, and adequacy of progestogen exposure should be reviewed before or alongside investigation. The 2025 European Society of Endocrinology guideline recommends reassessing symptom response and tolerability approximately 3 months after MHT initiation, with dose or formulation adjustment when response is inadequate or adverse effects occur, followed by periodic reassessment thereafter [9]. NICE guidance was further updated in April 2026 regarding unscheduled bleeding during systemic MHT [30]. Table 2 summarises the contemporary evidence for major clinical outcomes and highlights where formulation, route, and study design affect interpretation.

### Genitourinary Syndrome of Menopause: Treatment Selection and Special Considerations

GSM includes vulvovaginal dryness, burning or irritation, dyspareunia and other sexual symptoms, and urinary symptoms such as urgency, dysuria, and recurrent urinary tract infection. Treatment should be matched to the predominant symptom and severity rather than assuming that all genitourinary complaints have the same mechanism. Non-hormonal vaginal moisturisers are used regularly to improve baseline dryness, whereas lubricants are used primarily to reduce friction and pain during sexual activity; either can be used alone or together with local hormonal treatment [30]. Pelvic-floor assessment and therapy are appropriate when pelvic-floor overactivity, pain, urinary dysfunction, or musculoskeletal contributors coexist, and persistent dyspareunia warrants evaluation for dermatological, infectious, pelvic-floor, and other vulvovaginal causes.

For women without a history of breast cancer, low-dose vaginal estrogen has the most robust evidence for persistent GSM and can be delivered as cream, gel, tablet/pessary, or a vaginal ring according to preference, availability, and local labelling [30,31]. Systemic MHT is not required when GSM is the only indication. Vaginal prasterone (DHEA) is an option when vaginal estrogen or non-hormonal measures are ineffective or not tolerated, while oral ospemifene can be considered when local treatment is impractical or unsuitable [30]. Vaginal estrogen may also reduce recurrent urinary-tract infections in peri- and postmenopausal women and should be considered within the broader recurrent-UTI pathway rather than as systemic prophylaxis [30].

Energy-based vaginal laser and radiofrequency therapies should not be presented as established alternatives to approved GSM treatments. The 2025 AUA/SUFU/AUGS guideline concluded that current evidence does not support routine CO_2_ laser, Er:YAG laser, or radiofrequency treatment for GSM outcomes; if considered outside a trial for a woman who cannot use or declines established options, the experimental status and uncertainty regarding long-term efficacy and safety should be explicit [31].

## 5. Non-Hormonal Pharmacotherapy

For women who have a contraindication to systemic MHT, prefer not to use hormones, or have an inadequate response, several non-hormonal options are available. Neurokinin receptor antagonists target thermoregulatory signaling downstream of estrogen withdrawal without estrogenic or progestogenic activity. Other options, including selected SSRIs or SNRIs, gabapentin, and oxybutynin, act through different pathways and have distinct adverse-effect and interaction profiles.

**Fezolinetant (45 mg once daily).** Fezolinetant was approved by the FDA in May 2023 and received European Union marketing authorization in December 2023 [32]. In SKYLIGHT 1 and 2, placebo-adjusted reductions in moderate-to-severe VMS frequency were approximately 2 to 3 episodes per day at weeks 4 and 12 [33,34], with associated improvement in menopause-related quality of life in pooled analysis [35]. Post-marketing reports of serious liver injury led the FDA to add a boxed warning in December 2024. Baseline hepatic tests and follow-up testing monthly for the first 3 months and at months 6 and 9 are required in the United States [36]. Access differs by jurisdiction: in the United Kingdom, NICE technology appraisal guidance issued in 2026 recommends fezolinetant as an option for moderate-to-severe VMS when MHT is unsuitable [37].

**Elinzanetant (120 mg once daily at bedtime).** Elinzanetant was approved by the FDA in October 2025 as a dual NK1 and NK3 receptor antagonist. In OASIS 1 and 2, placebo-adjusted mean reductions in VMS frequency at week 12 were approximately 3.2 episodes per day, with improvements in sleep disturbance and menopause-related quality of life [38]. OASIS 3 reported a smaller placebo-adjusted effect in a broader population and provided 52-week safety data [39]. The US label requires baseline hepatic testing and repeat transaminase assessment at 3 months; moderate-to-severe hepatic impairment is not recommended [40]. In OASIS 4, women with VMS related to endocrine therapy for hormone-receptor-positive breast cancer or risk reduction had a week-12 least-squares mean difference of −3.4 moderate-to-severe VMS episodes per day versus placebo (95% CI −4.2 to −2.5) [41]. No head-to-head trial has compared elinzanetant with fezolinetant. Differences between numerical effect estimates from separate trials must not be interpreted as evidence that one agent is more effective, because trial populations, baseline symptom burden, protocols, estimands, and placebo responses differ. OASIS 4 provides evidence for VMS efficacy in an oncology population but does not establish long-term oncological safety or breast-cancer recurrence outcomes. Table 3 compares the two approved neurokinin-targeted therapies using similar outcome metrics and current regulatory monitoring requirements.

Cendifensine (NOE-115) is an investigational monoamine reuptake modulator in phase 2 development. Preliminary phase 2a results reported substantial reductions from baseline in VMS frequency and possible effects on mood, appetite, and fatigue, but these findings have not yet been confirmed in a large peer-reviewed randomised phase 3 trial. The agent is therefore discussed only as an emerging therapy [42].

**SSRIs and SNRIs.** Paroxetine, venlafaxine, desvenlafaxine, escitalopram, and citalopram are antidepressant drugs that have evidence for reducing VMS, although effect sizes are generally smaller than with systemic MHT [43]. Low-dose paroxetine is FDA-approved for VMS; other agents are frequently used off-label. Paroxetine and fluoxetine strongly inhibit CYP2D6 and are generally avoided with tamoxifen because they can reduce formation of its active metabolite, whereas venlafaxine and citalopram or escitalopram have less CYP2D6 inhibition.

Gabapentin, oxybutynin, and testosterone. Gabapentin is an anticonvulsant that can reduce VMS and may be useful when nocturnal symptoms or sleep disturbance predominate, although dizziness and somnolence can limit use. Oxybutynin (an anticholinergic drug primarily used for overactive bladder) can reduce VMS in randomized placebo-controlled trials, including in women with breast cancer, but its anticholinergic adverse-effect profile warrants caution in older women or those with cognitive risk [44]. GSM treatment is discussed separately in Section Genitourinary Syndrome of Menopause: Treatment Selection and Special Considerations. Systemic transdermal testosterone has evidence for postmenopausal hypoactive sexual desire disorder after biopsychosocial assessment, but it is not a general treatment for fatigue, cognition, or wellbeing and is off-label in many countries [45].

**Medication review and interactions.** Interaction risk is a recurring determinant of treatment selection across hormonal and non-hormonal care. Table 4 summarizes selected high-impact interactions that are most likely to change prescribing decisions; it is deliberately not exhaustive and should be interpreted alongside current product information.

## 6. Microbiome, Phytoestrogens, and the Boundary of Precision Medicine

**Figure 2:**
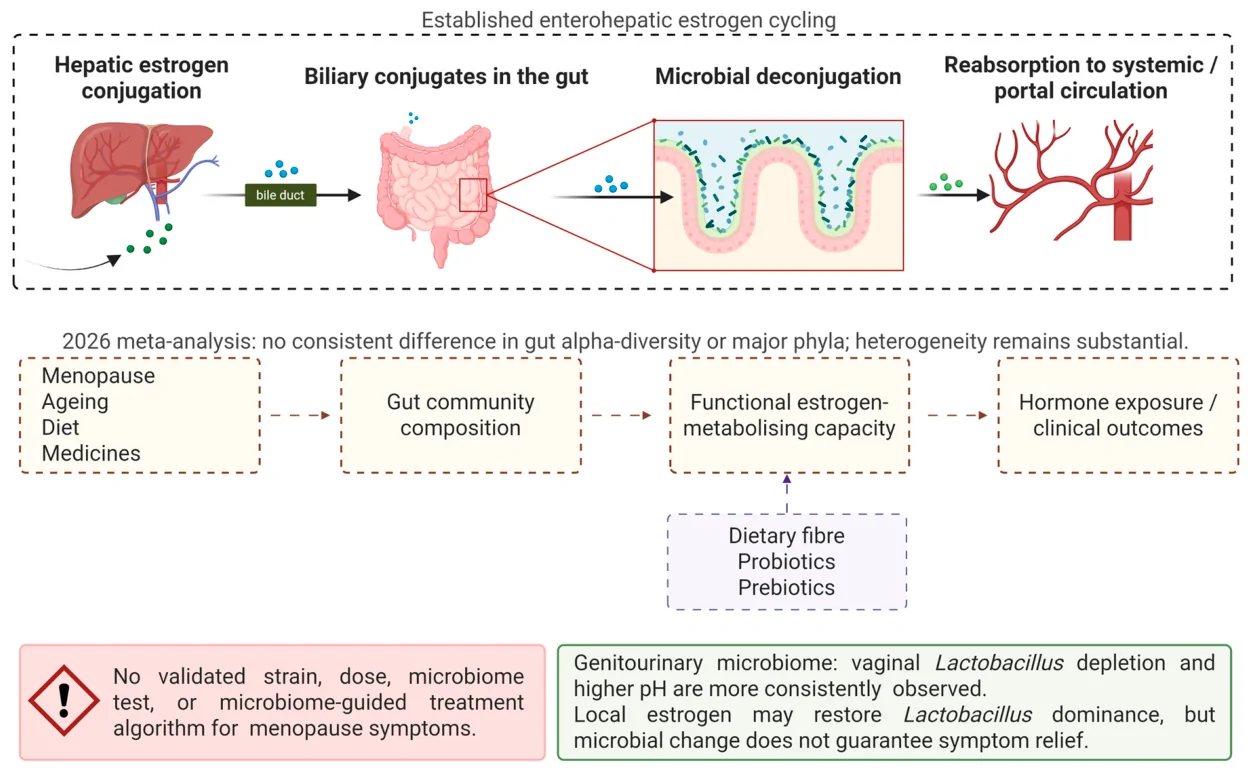
Evidence-aware model of the estrobolome and microbiome–endocrine interface. Solid arrows denote established biochemical steps in enterohepatic estrogen metabolism; dashed arrows denote proposed, associative, or not-yet-validated clinical links. The upper frame encloses the established enterohepatic pathway; cream boxes show the proposed gut microbiome–estrogen pathway; the lavender box shows candidate dietary, prebiotic, and probiotic interventions; the red box summarises current evidence gaps; and the green box summarises genitourinary microbiome findings. Current meta-analytic evidence does not establish a universal postmenopausal gut microbiome signature, whereas vaginal *Lactobacillus* depletion is more consistently observed in GSM. Microbiome restoration does not reliably predict symptom resolution, and no microbiome-directed treatment-selection algorithm is validated [47,48,49,50,51]. Created in BioRender.com.

The estrobolome describes the aggregate microbial genes and enzymes capable of metabolising estrogens, particularly through deconjugation of biliary estrogen metabolites [46,47,48]. The biochemical mechanism is plausible and experimentally demonstrable, but the clinically relevant magnitude of microbiome-mediated estrogen recycling in humans remains uncertain. This section therefore uses the microbiome and phytoestrogen literature as a test case for the boundary between mechanism-based personalization and validated precision medicine: evidence is separated into mechanistic, observational, interventional, and treatment-selection levels (Figure 2).

### 6.1. Gut Microbiome and the Estrobolome

**Biochemical foundations.** Estrogens produced by the ovaries and peripheral tissues undergo hepatic metabolism and conjugation before biliary or urinary excretion. In the intestine, microbial β-glucuronidases and other enzymes can deconjugate selected metabolites, permitting reabsorption [47,48]. This mechanism is established biochemically, but its quantitative importance varies by substrate, enzyme class, microbial community, intestinal transit, medication exposure, and host metabolism.

Experimental evidence demonstrates that specific microbial β-glucuronidases can reactivate estrogen glucuronides [47]. Reviews have identified additional microbial enzymes potentially involved in estrogen metabolism [48]. These observations support the concept of a microbiome–endocrine interface, but they do not by themselves demonstrate that changing microbiome composition improves clinical menopause outcomes.

**Microbiome changes across menopause.** Cross-sectional studies have reported differences in microbial composition between premenopausal and postmenopausal groups, but findings vary by age, adiposity, diet, medication use, geography, sequencing method, and analytic pipeline [52]. Importantly, a 2026 systematic review and meta-analysis of seven studies found no significant difference in gut alpha-diversity, Bacteroidetes, Firmicutes, or the Bacteroidetes-to-Firmicutes ratio between hypoestrogenic and euestrogenic groups, with substantial heterogeneity across studies [49]. No universal postmenopausal gut microbiome signature has therefore been established, and individual taxa or diversity scores should not be interpreted as diagnostic or treatment-selection biomarkers.

**Proposed systemic consequences and unresolved mechanisms.** Reduced short-chain-fatty-acid production and impaired intestinal-barrier function are plausible links between dysbiosis, inflammation, and cardiometabolic risk, but human studies mainly demonstrate associations rather than causality. Estrogen exposure can in turn influence microbial ecology, while microbial metabolism can affect the pool of deconjugated estrogen metabolites; evidence for a self-reinforcing clinical feedback loop between MHT, microbial beta-glucuronidase activity, and systemic estrogen concentrations remains preliminary and is better presented as a testable model than as a confirmed therapeutic mechanism. Common medicines can also alter gut microbial composition, and microbial enzymes can modify the disposition of some drugs and endogenous compounds, but in menopause care this remains an emerging research question rather than an established explanation for interindividual variation in MHT response. Large, well-controlled longitudinal cohorts, prospective pharmacokinetic studies, and intervention trials are needed to determine whether microbiome changes contribute independently to menopause symptoms or primarily reflect ageing and related exposures, and are required before microbiome profiles could guide prescribing.

**Microbiome-directed interventions.** A small randomised trial reported that a probiotic formulation with beta-glucuronidase activity altered serum estrogen measures in peri- and postmenopausal women [53]. However, proof of a biochemical effect is not proof of symptom benefit or of a clinically useful treatment-selection strategy. A 2026 systematic review of *Lactobacillus*-based interventions for GSM identified only nine studies with 751 participants and found limited, heterogeneous evidence for lower urinary-tract and vaginal outcomes [51]. Current evidence does not establish a strain, dose, duration, target phenotype, or routine menopause indication for probiotic prescribing. Dietary fibre supports microbial diversity and short-chain-fatty-acid production and should be encouraged for general cardiometabolic and gastrointestinal health, but claims that specific prebiotics reliably restore an estrogen-supportive microbiome remain unproven. Faecal microbiota transplantation is not an established treatment for menopause symptoms and should remain confined to approved indications or research protocols.

### 6.2. Genitourinary Microbiome

Evidence is more consistent for the genitourinary than for the gut microbiome. Declining estrogen reduces epithelial glycogen and is commonly associated with higher vaginal pH, reduced *Lactobacillus* dominance, and greater microbial diversity. A 2026 systematic review of 23 studies involving 5027 participants found consistent postmenopausal *Lactobacillus* depletion and greater microbial diversity across genitourinary studies, although associations between specific community states and individual GSM symptoms were weaker and heterogeneous [50].

Low-dose vaginal estrogen improves GSM and can partially restore a more *Lactobacillus*-dominant environment, but microbiome restoration does not uniformly predict clinical symptom resolution [50]. Vaginal probiotics have variable and limited evidence and should not substitute for established GSM therapy [51]. Next-generation sequencing remains valuable in research but is not routinely recommended for diagnosis or treatment selection in postmenopausal women [50,54]. Gut and vaginal microbiome findings should therefore not be conflated: the direction and clinical meaning of diversity differ by anatomical site.

### 6.3. Phytoestrogens and Equol Metabolism

**Chemistry and receptor activity.** Isoflavones, lignans, and coumestans are plant-derived compounds that can interact with estrogen receptors and other signalling pathways. Genistein generally has greater affinity for ERβ than ERα, but receptor-binding observations do not translate directly into tissue-selective clinical safety or efficacy. Preparation, dose, metabolism, and baseline diet contribute to heterogeneous trial results [55,56,57]. Isoflavones include genistein, daidzein, and glycitein from soy and related compounds from red clover. Lignans from flaxseed and sesame are converted by gut bacteria to enterolignans. Daidzein can be converted to S-equol by specific microbial communities, creating a measurable metabolic phenotype that may influence exposure to active metabolites.

**Equol-producing status.** Approximately 30–50% of adults in many Western cohorts and a higher proportion in some Asian cohorts produce measurable equol after soy or daidzein exposure [55]. Producer status is better described as a pharmacomicrobiomic or microbiome-dependent metabolic phenotype than as a pharmacogenomic variable. Some studies suggest differential responses to isoflavones, but results are inconsistent and do not yet validate equol testing as a routine treatment-selection tool. Urinary equol measurement after a standardised soy or daidzein challenge and metagenomic detection of equol-producing pathways are research tools. Non-producers may still respond to genistein or other isoflavones through equol-independent mechanisms. Preformed S-equol products are available in some markets, but comparative effectiveness and long-term safety data remain insufficient to support a universal phenotype-guided algorithm.

**Clinical evidence.** A 2025 systematic review and meta-analysis of 12 randomised trials found a small improvement in overall menopausal symptom scores with soy isoflavones, but no significant treatment effect on hot flushes, excessive sweating, insomnia, or the vasomotor-symptom domain [56]. A separate 2024 meta-analysis of five RCTs involving 425 women found no significant improvement in overall menopausal symptoms or quality of life, although depression scores improved [58]. These findings support, at most, modest and domain-specific effects rather than use of soy isoflavones as an evidence-based substitute for MHT or approved neurokinin antagonists for VMS. Preparation, dose, adherence, background diet, and metabolic phenotype remain important sources of heterogeneity. Trials have also examined bone density, lipids, glucose metabolism, vascular function, mood, and cognition, but findings are inconsistent and generally insufficient to establish phytoestrogen supplements as alternatives to osteoporosis, cardiovascular, or neurocognitive therapies; mechanistic and preclinical findings should be separated from clinical outcomes. The main phytoestrogen classes, their mechanisms, human evidence, and limitations are summarised in Table 5.

**Safety and product quality.** Dietary soy foods are generally considered safe for most women, including many breast-cancer survivors, whereas concentrated supplements require greater caution because dose, purity, labelling, and interactions vary. EFSA previously found no signal of harm to breast, thyroid, or uterine tissue at commonly studied supplemental doses in postmenopausal women [57]. Reassuringly, a 2025 meta-analysis of 40 randomized trials (3285 participants) found no statistically significant effect of soy isoflavones on endometrial thickness, vaginal maturation index, FSH, or circulating estradiol [59]. These data address selected measures of estrogenicity but do not eliminate the need for product-quality assessment or oncology-specific clinical judgement.

**Mediterranean-style dietary pattern.** A dietary pattern rich in vegetables, legumes, whole grains, nuts, olive oil, fish, and minimally processed foods supports cardiovascular, metabolic, and bone health. It also supplies fibre and polyphenols that influence microbial ecology. Evidence for direct reduction of VMS is less consistent than evidence for broader health benefits; dietary counselling should therefore emphasise overall health, adequate protein and calcium, and sustainable weight management rather than promising symptom-specific effects [60].

## 7. Behavioural, Mind–Body, and Lifestyle Interventions

Behavioural and lifestyle interventions are important components of comprehensive menopause care, but their effects must be reported by outcome rather than treated as a single category. An intervention can improve symptom bother, sleep, mood, physical function, bone health, or cardiometabolic risk without materially reducing physiological VMS frequency. This distinction is central to avoiding overstatement and is summarised in Table 6 [43,61].

**Cognitive behavioural therapy, including CBT for insomnia.** Menopause-specific CBT reduces VMS-related bother, interference, anxiety, and sleep disturbance even when objective hot-flash frequency changes little [30,62]. CBT is therefore complementary to pharmacological treatment rather than a direct substitute for the thermoregulatory effects of MHT or neurokinin antagonists. CBT-I has specific evidence for menopausal insomnia rather than for direct suppression of VMS. A 2025 systematic review and meta-analysis of 11 RCTs involving 973 women found improvements in sleep quality and insomnia severity across face-to-face, telephone, and internet-delivered formats [63]. CBT-I should therefore be considered a targeted treatment for insomnia in peri- and postmenopausal women, while nocturnal VMS and other contributors are managed in parallel.

**Clinical hypnosis.** A 2025 scoping review identified 23 studies and concluded that clinical-hypnosis studies reported reductions in hot-flash frequency and severity, whereas CBT studies more consistently improved bother and interference [64]. The evidence base remains smaller than that for established pharmacological treatments, and comparisons across interventions are indirect. A pivotal randomised trial reported a 74% reduction in hot-flash scores with clinical hypnosis compared with 17% with structured attention control [65]. These findings are promising, but they should not be interpreted as demonstrating equivalence to fezolinetant or elinzanetant because populations, outcomes, placebo controls, and trial designs differ. Access to trained practitioners and the need for repeated sessions remain practical limitations.

**Mindfulness, yoga, and relaxation.** These interventions may be useful for stress, mood, physical function, flexibility, or subjective wellbeing in selected women, but this should be separated from claims of VMS treatment. The 2023 North American Menopause Society position statement did not recommend exercise, yoga, mindfulness-based interventions, relaxation, cooling techniques, trigger avoidance, soy/equol, or acupuncture as evidence-based treatments for reducing VMS because results were negative, inconsistent, or insufficient [43]. They can still be offered for other appropriate health goals when expectations are explicit and patient preference supports their use.

**Physical activity and structured exercise.** Regular aerobic activity and resistance training should be actively encouraged for cardiometabolic health, maintenance of muscle and physical function, psychological wellbeing, and bone-health support. These benefits are clinically important in midlife and after menopause, but exercise should not be presented as an established treatment for reducing hot-flash frequency; the 2023 North American Menopause Society statement did not recommend it for that specific indication [43,61]. Resistance training deserves particular emphasis because preservation of lean mass and strength is a meaningful menopause-health goal independent of VMS response.

**Weight management and anti-obesity pharmacotherapy.** Weight reduction in women with overweight or obesity may reduce VMS and improve cardiometabolic risk, sleep apnoea, mobility, and several cancer-related risk factors. GLP-1 or dual GIP/GLP-1 receptor agonists should be prescribed according to established obesity or diabetes indications rather than as menopause treatments. A 2026 retrospective cohort found greater tirzepatide-associated weight loss among MHT users than non-users, but residual confounding prevents causal interpretation and randomised studies are required [66].

**Sleep disturbance and behavioral sleep treatment.** Sleep problems during the menopausal transition are heterogeneous and may reflect VMS-related awakenings, insomnia, depression or anxiety, obstructive sleep apnoea, restless legs, medicines, alcohol, pain, or other contributors. Management should therefore identify the dominant mechanism rather than assuming that all sleep disturbance is caused directly by estrogen withdrawal. CBT-I has the strongest targeted behavioural evidence for menopausal insomnia [63]. MHT can improve sleep when nocturnal VMS are a major driver, and elinzanetant improved sleep outcomes in VMS trials, but it should not be described as a treatment for primary insomnia in the absence of VMS. Screening and treatment for sleep apnoea, mood disorders, and other sleep disorders remain important.

**Acupuncture.** Acupuncture studies for menopausal VMS are difficult to interpret because sham controls can have substantial contextual and physiological effects, and real acupuncture has not consistently outperformed sham treatment for hot-flash frequency. Pragmatic comparisons with waiting-list or no-treatment controls may show patient-reported improvement, but these designs do not establish a specific VMS effect. The 2023 North American Menopause Society position statement therefore classified acupuncture as not recommended for VMS treatment on the available evidence [43]. It may still be chosen for other indications or as a patient-preferred complementary intervention when expectations, cost, and evidence uncertainty are discussed.

**Dietary and environmental strategies.** Alcohol, caffeine, spicy foods, heat, and other exposures are reported as triggers by some women, but controlled evidence does not support universal avoidance as a treatment for VMS [43]. Individual symptom diaries can be useful when a reproducible trigger is suspected, and cooling or layered clothing may improve comfort without being described as disease-modifying therapy. Calcium and vitamin D intake should be assessed according to diet, fracture risk, and local osteoporosis guidance; routine high-dose supplementation for all postmenopausal women is not supported.

## 8. Special Populations

Menopause management should not be approached as a uniform clinical entity. Premature ovarian insufficiency, treatment-induced menopause, breast-cancer endocrine therapy, surgical menopause, and major differences in access to care require modified risk–benefit assessment and coordinated management.

### 8.1. Premature Ovarian Insufficiency

Premature ovarian insufficiency (POI), defined by loss of ovarian activity before age 40, occurs in at least 1–3% of women in contemporary guidance and differs fundamentally from natural menopause because of the longer duration of estrogen deficiency and its implications for bone, cardiovascular, sexual, reproductive, and psychological health [9,67]. Causes include genetic, autoimmune, iatrogenic, and idiopathic forms. Diagnosis and treatment should follow current POI-specific guidance rather than routine natural-menopause pathways.

In the absence of a contraindication, physiological-dose hormone replacement is generally recommended until approximately the usual age of natural menopause [67]. Regimens and doses should be individualised, and adequate endometrial protection is required for women with a uterus. Fertility counselling, bone-density assessment, cardiovascular risk management, psychological support, and evaluation for associated genetic or autoimmune conditions may be required. AMH can contribute to assessment after gonadotoxic treatment but should not be used as the sole determinant of long-term outcomes [68].

### 8.2. Menopause in Breast-Cancer Survivors

Systemic MHT is generally not recommended in women with a previous breast cancer diagnosis, particularly after hormone-receptor-positive disease [57]. Exceptional cases with severe refractory symptoms may require multidisciplinary shared decision-making after non-hormonal options have failed. OASIS 4 provides important contemporary evidence: among women with moderate-to-severe VMS related to endocrine therapy for hormone-receptor-positive breast cancer or risk reduction, elinzanetant produced a week-12 placebo-adjusted least-squares mean difference of −3.4 episodes per day (95% CI −4.2 to −2.5) [41]; this efficacy result should not be interpreted as evidence of long-term oncological safety. Venlafaxine, citalopram, escitalopram, gabapentin, CBT, and clinical hypnosis may also be considered according to symptom phenotype and drug interactions. For GSM, moisturisers and lubricants are first-line. When symptoms remain clinically important, low-dose vaginal estrogen can be considered after individual risk–benefit discussion, but endocrine therapy matters: use alongside tamoxifen is generally considered less concerning mechanistically because tamoxifen blocks the estrogen receptor in breast tissue, whereas women receiving aromatase inhibitors require more cautious multidisciplinary shared decision-making because exogenous estrogen is not receptor-blocked and transient systemic absorption may be relevant [9,30,69]. Vaginal DHEA or other prescription options may be considered when vaginal estrogen is unsuitable, but local labelling, oncology guidance, and the limited long-term safety evidence should be reviewed [69].

### 8.3. Surgical Menopause

Surgical menopause following bilateral oophorectomy differs fundamentally from natural menopause because ovarian hormone production ceases abruptly rather than declining progressively over several years. When bilateral oophorectomy is performed before the usual age of natural menopause, women may experience more severe vasomotor symptoms, sleep disturbance, mood changes, sexual dysfunction, and genitourinary symptoms. Earlier and prolonged estrogen deprivation is also associated with adverse skeletal and cardiometabolic consequences and may contribute to cognitive and neurological outcomes, although the magnitude of these risks varies according to age at surgery, baseline health, indication for surgery, and subsequent use of hormone therapy. For women without contraindications, systemic MHT is generally recommended after premenopausal bilateral oophorectomy and continued at least until approximately the average age of natural menopause. In this context, treatment should be understood as physiological hormone replacement rather than solely as short-term symptom management. Women with an intact uterus require adequate endometrial protection with a progestogen, whereas estrogen-only therapy can generally be used after hysterectomy. Dose and route should be individualised according to symptom control, age, cardiovascular and thrombotic risk, comorbidities, and patient preference. Women undergoing risk-reducing salpingo-oophorectomy because of BRCA1 or BRCA2 pathogenic variants require particularly careful counselling. In carriers without a personal history of hormone-receptor-positive breast cancer, available evidence suggests that short-term MHT after risk-reducing surgery does not negate the reduction in breast-cancer risk achieved by oophorectomy [70]. However, the choice of regimen should take account of uterine status, previous cancer history, residual breast-cancer risk, and the age at surgery. Decisions in women with a previous breast cancer diagnosis should be made collaboratively with the oncology team because systemic MHT is generally avoided, particularly after hormone-receptor-positive disease.

Preoperative counselling is essential and should include the likely symptom burden, fertility implications where relevant, bone health, cardiovascular risk, sexual function, psychological wellbeing, and available hormonal and non-hormonal treatments. Baseline assessment may include cardiovascular risk factors, bone-health history, medication use, and symptoms affecting sexual health. Follow-up should evaluate treatment efficacy, adherence, bleeding patterns, genitourinary symptoms, blood pressure, weight and metabolic risk, bone-health needs, and changes in individual treatment goals. Surgical menopause should therefore be managed as a planned longitudinal clinical pathway rather than as an incidental postoperative consequence.

### 8.4. Previous Venous Thromboembolism or Thrombophilia

A history of VTE or thrombophilia does not create a uniform management pathway. If systemic MHT remains indicated after individualized assessment, oral estrogen should generally be avoided because of its greater thrombotic effect; the 2025 European Society of Endocrinology guideline recommends low-dose transdermal estrogen after individualized risk–benefit assessment in women with previous VTE [9]. Specialist input is appropriate for complex thrombophilia, recurrent events, active anticoagulation, or uncertainty about baseline risk. Endometrial protection remains necessary when the uterus is present.

### 8.5. Obesity, Metabolic Syndrome, and Cardiometabolic Comorbidity

Obesity, insulin resistance, diabetes, hypertension, dyslipidaemia, and sleep apnoea frequently cluster around midlife and can influence both symptom burden and treatment choice. These conditions are not automatically contraindications to MHT when adequately controlled, but they increase the importance of cardiovascular-risk assessment, weight management, blood-pressure control, and medication review. For women with obesity or intermediate thrombotic or cardiovascular risk who otherwise have an indication for MHT, transdermal estradiol is generally preferred to oral estrogen because it avoids first-pass hepatic exposure [9]. Anti-obesity pharmacotherapy should be used for established obesity indications rather than as menopause-specific treatment.

### 8.6. Endometriosis and Surgical Menopause

Women with a history of endometriosis require additional consideration after natural or surgical menopause because residual disease can remain hormonally responsive. ESHRE guidance supports consideration of combined MHT for symptomatic women with a history of endometriosis and advises against estrogen-only regimens for vasomotor symptoms because of concern about reactivation or malignant transformation of residual disease [71]. After early surgical menopause for endometriosis, combined estrogen-progestogen therapy is generally continued at least until the usual age of natural menopause when not contraindicated. Persistent or recurrent pelvic pain or a new pelvic mass after menopause warrants reassessment rather than attribution to menopause alone.

### 8.7. Ethnic, Socioeconomic, and Access Disparities

The menopause evidence base continues to underrepresent many racial and ethnic groups, women from lower-income populations, rural communities, migrants, and women with limited access to specialist care. This affects both the generalisability of clinical evidence and the ability of guidelines to address differences in symptom experience, treatment preferences, comorbidity patterns, healthcare access, and medication affordability. Reported differences in VMS duration, severity, sleep, mood, and quality of life are unlikely to have a single explanation. A 2026 systematic review and meta-analysis found associations between VMS patterns and factors including ethnicity, smoking, obesity, depression, education, and income [3]. These findings should be interpreted within social, behavioural, biological, environmental, and health-care contexts rather than as deterministic population biology. Differences should not be attributed primarily to genetics, soy consumption, equol production, or microbiome composition without direct supporting evidence. Access disparities can occur at several stages of care. Some women may not recognise their symptoms as part of the menopausal transition, while others may encounter clinicians with insufficient training or reluctance to discuss MHT. Language barriers, consultation time, cost, fragmented care, and the absence of locally available menopause specialists may further restrict treatment choices. Even when treatment is offered, inequities in access to transdermal preparations, newer non-hormonal medications, pelvic-floor care, psychological therapies, and specialist oncology consultation may prevent genuinely individualised management.

Future studies should recruit populations that better reflect the diversity of women affected by menopause and should report ethnicity, socioeconomic position, geographic context, health literacy, disability, comorbidities, and access to care. Investigators should also examine whether treatment effects, adverse events, adherence, and treatment preferences differ across population groups. At a service level, improving menopause care requires accessible evidence-based information, clinician education, culturally appropriate communication, shared decision-making, and affordable treatment pathways. Equity should be considered an essential dimension of treatment effectiveness rather than a separate public-health concern.

## 9. From Personalized Care to Precision Menopause Medicine

The next stage of menopause research is likely to involve both improved application of established treatments and development of new therapeutic approaches. Experimental strategies targeting ovarian ageing include modulation of the AMH pathway, mTOR-related signalling, mitochondrial dysfunction, cellular senescence, ovarian inflammation, and follicular activation. Ovarian-tissue cryopreservation and reimplantation may have roles in selected women at risk of iatrogenic ovarian insufficiency. However, most approaches intended to preserve ovarian reserve or delay menopause remain preclinical or in early clinical development. Claims that they can delay natural menopause by a defined number of years are not currently supported by clinical outcome trials [7]. Drug-delivery research is also exploring local, tissue-targeted, nanoparticle-based, and controlled-release hormone formulations. Such approaches could theoretically improve local efficacy or reduce systemic exposure, particularly in GSM. Nevertheless, evidence that these systems can reliably produce beneficial effects in bone, brain, cardiovascular, or urogenital tissues while completely avoiding breast or endometrial exposure is not yet available. Future studies must assess clinically meaningful outcomes rather than relying only on pharmacokinetic, cellular, or animal-model findings. Neurokinin-pathway therapeutics will probably remain an active area of development. Priorities include longer follow-up, direct comparisons between agents, studies in women with cancer and complex comorbidity, identification of clinically significant drug interactions, and evaluation of effects beyond VMS, including sleep, mood, cognition, and quality of life. Comparative-effectiveness studies are also required to establish how these agents perform against MHT, SSRIs or SNRIs, gabapentin, and behavioural treatments in routine clinical practice. Multi-omic profiling may eventually identify clinically useful subgroups based on drug metabolism, symptom biology, inflammatory pathways, or microbiome-derived metabolic phenotypes. Candidate markers include equol-producing status, pharmacokinetic variants, microbial estrogen-metabolising capacity, and biomarkers of neurokinin signalling [72]. However, these variables have not been prospectively validated as routine predictors of response to MHT, neurokinin antagonists, phytoestrogens, or other menopause treatments. The key research transition is therefore from association to test-guided utility: a biomarker must do more than correlate with symptoms or drug exposure.

**Figure 3:**
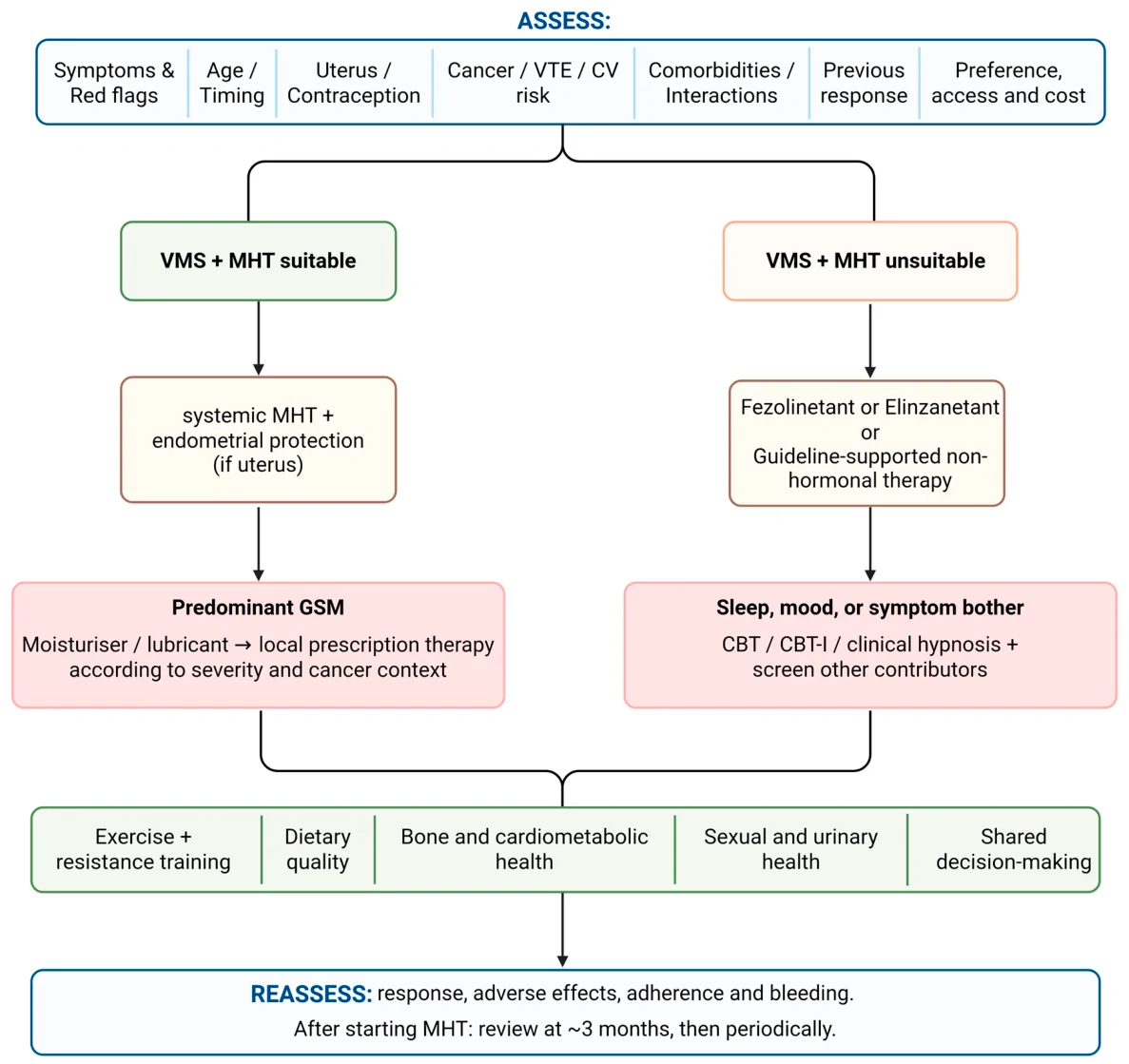
Practical clinical-actionability framework for treatment selection. Current evidence-based personalisation is principally clinical rather than omic. The pathway incorporates symptom phenotype, uterine and reproductive status, age/timing, contraindications, comorbidities, cancer and thrombotic risk, interactions, access, and preference; after MHT initiation, response and tolerability should be reassessed at approximately 3 months and periodically thereafter [9,13,14,24,30,43]. Solid arrows and connecting lines show the sequence of clinical decisions. CBT, cognitive behavioural therapy; CBT-I, CBT for insomnia; CV, cardiovascular; GSM, genitourinary syndrome of menopause; MHT, menopausal hormone therapy; VMS, vasomotor symptoms; VTE, venous thromboembolism. Created in BioRender.com.

A biomarker becomes clinically useful only when it adds information beyond standard clinical assessment, changes the treatment selected, improves meaningful patient outcomes, and is sufficiently reliable, reproducible, accessible, and cost-effective. Precision menopause medicine should therefore not be equated simply with more testing. In 2026, the most evidence-based form of personalisation remains clinical assessment of dominant symptoms, age and time since menopause, uterine and reproductive status, contraindications, cancer and thrombotic risk, comorbidities, medicines, previous treatment response, affordability, access, and informed preference (Figure 3). Microbiome sequencing, equol testing, and multi-omic profiling remain promising research tools, not routine prerequisites for personalised care. Table 7 summarises the clinical-actionability boundary for unsupported, emerging, and experimental strategies that are not captured in the intervention-specific tables above.

## 10. Research Gaps

Several important gaps limit the certainty and generalisability of current menopause management. First, longer-term comparative safety and effectiveness data are needed for neurokinin receptor antagonists. Direct head-to-head trials should compare fezolinetant and elinzanetant using standardised VMS outcomes, sleep measures, quality-of-life instruments, adverse-event definitions, and follow-up periods. Studies should include older women and women with cardiovascular, hepatic, renal, oncological, and psychiatric comorbidities who may be underrepresented in registration trials. Second, MHT research must provide more formulation-specific evidence. Oral and transdermal estrogen, micronised progesterone, dydrogesterone, synthetic progestins, and different treatment schedules should not be treated as a single homogeneous exposure. Further research is needed on absolute rather than only relative risks, management of unscheduled bleeding, duration of treatment, discontinuation strategies, use after age 60, and outcomes associated with prolonged treatment. Greater representation of ethnically, socially, and clinically diverse populations is essential.

Third, the causal contribution of gut and vaginal microbiomes remains uncertain and should be studied separately by anatomical site. The 2026 gut meta-analysis highlights heterogeneity and the absence of a consistent global gut-diversity signal [49], while the genitourinary literature shows a more reproducible pattern of *Lactobacillus* depletion without a reliable one-to-one relationship between microbiome restoration and symptom resolution [50]. Future studies require longitudinal designs, standardised sampling and sequencing, control of diet and medicines, functional rather than taxonomic endpoints where appropriate, and prespecified clinical outcomes. Probiotic trials should identify strains precisely and demonstrate symptom benefit, not merely microbial change [51]. Routine microbiome testing should not be recommended until test-guided management improves outcomes compared with clinical assessment alone. Fourth, phytoestrogen studies require better standardisation of product composition, dose, bioavailability, treatment duration, background diet, adherence, equol-producing phenotype, and adverse-event reporting. Trials should differentiate whole-food interventions from isolated supplements and should avoid generalising results obtained with one formulation to the entire phytoestrogen category. Fifth, behavioural and lifestyle trials require larger and more representative samples, appropriate controls, validated outcome instruments, and sufficient follow-up. Future studies should distinguish objectively or prospectively recorded VMS frequency from symptom bother, sleep, coping, mood, physical function, bone or muscle outcomes, and quality of life. This separation is essential because an intervention can be clinically worthwhile without being a thermoregulatory VMS treatment. Implementation research is also needed to determine how effective interventions such as CBT, CBT-I, and clinical hypnosis can be delivered affordably and at scale [43,63,64].

Finally, biomarker and multi-omic strategies require prospective validation. Studies should demonstrate not only an association with symptoms or treatment response, but also that using the biomarker to select treatment produces better outcomes than clinical assessment alone. Cost-effectiveness, reproducibility, acceptability, and access must be evaluated before such approaches can be incorporated into routine menopause care.

## 11. Conclusions

Menopause care now includes a wider range of hormonal, non-hormonal, behavioural, and supportive interventions than previously, but the evidence is not equally mature across domains. Systemic MHT remains the most effective treatment for VMS in appropriately selected women and contributes to prevention of early postmenopausal bone loss. Local vaginal therapies are central to GSM management. Treatment selection should distinguish natural menopause from POI, surgical menopause, cancer-related menopause, and clinically important comorbidities, and should incorporate the 2025–2026 guideline and regulatory updates described in this review [9,17,30].

Fezolinetant and elinzanetant provide mechanism-based non-hormonal alternatives, particularly for women who cannot or do not wish to use MHT. Their efficacy should be communicated using comparable placebo-adjusted outcomes rather than unadjusted percentage reductions, and their hepatic precautions, monitoring requirements, adverse effects, and drug interactions must be considered. Existing options including SSRIs or SNRIs, gabapentin, oxybutynin, CBT, clinical hypnosis, exercise, sleep interventions, and weight management retain important roles according to the principal symptoms and individual clinical context. The gut microbiome, vaginal microbiome, estrobolome, and phytoestrogen metabolism provide biologically plausible avenues for future stratification, but plausibility should not be mistaken for clinical utility. Current evidence does not support routine microbiome sequencing, equol testing, targeted probiotic prescribing, or FMT for menopause symptoms [49,50,51]. Recent soy meta-analyses support, at most, modest and heterogeneous symptom-domain effects and do not establish soy isoflavones as a VMS therapy [56,58]; reassuring data on selected estrogenicity markers do not change that efficacy conclusion [59].

Personalised menopause care should therefore distinguish clearly between established treatment, evidence-supported extrapolation, emerging human evidence, and experimental strategies. The main actionable form of personalisation today is clinical rather than omic: symptom priorities, age and timing, uterine and reproductive status, contraindications, comorbidities, cancer and thrombotic risk, medication interactions, previous response, access, cost, and informed preference. Periodic reassessment is essential because symptoms, health risks, treatment availability, and patient goals change over time. The next scientific milestone for precision menopause medicine will not be the discovery of more associations, but prospective evidence that a biomarker-guided decision improves outcomes compared with careful clinical assessment alone.

## Figures and Tables

**Table 1:** Simplified STRAW+10 staging of reproductive ageing. Menstrual-cycle criteria are primary; hormonal measurements are supportive and may fluctuate. Stage +1c remains within early postmenopause; stage +2 denotes late postmenopause [8].

Stage	Name	Supportive Hormonal Features	Clinical Characteristics
–3b/–3a	Late reproductive	AMH and inhibin B ↓; FSH usually remains in the premenopausal range	Regular cycles; fertility possible; VMS uncommon
–2	Early transition	FSH becomes variable and may rise; AMH is ↓	Persistent difference of ≥7 days in the length of consecutive cycles; first VMS may appear; mood changes
–1	Late transition	FSH is frequently ↑ and estradiol fluctuates; AMH is ↓↓	Cycles ≥60 days; VMS frequent; sleep disruption; contraception still needed
+1a/+1b	Early postmenopause	FSH remains elevated and estradiol is generally ↓	VMS typically peak; rapid bone loss begins; GSM onset
+1c	Early postmenopause	FSH remains elevated; estradiol is generally ↓	Early postmenopausal phase; VMS may persist; bone loss is prominent
+2	Late postmenopause	Stable ↓ estradiol with persistently elevated gonadotropins	GSM and age-related bone and cardiometabolic risks become increasingly important

AMH = anti-Müllerian hormone; FSH = follicle-stimulating hormone; GSM = genitourinary syndrome of menopause; VMS = vasomotor symptoms; ↑ increased; ↓ low/decreased; ↓↓ markedly decreased.

**Table 2:** Contemporary MHT evidence by outcome, with clinical-actionability tiers as defined in Section 2.4. Tiers describe readiness for the stated clinical use, not the methodological quality of individual studies.

Outcome	Evidence Summary	Route/Formulation Considerations	Evidence Base	Tier	Clinical Interpretation
Vasomotor symptoms	Most effective symptom reduction	Systemic estrogen; protect endometrium if uterus present	RCTs/guidelines	A	First-line for eligible women with bothersome VMS
Genitourinary syndrome	Improves dryness, dyspareunia and urinary symptoms	Low-dose local estrogen; formulation by preference/availability	RCTs/guidelines	A	Prefer local therapy when GSM is the only indication
Bone	Prevents bone loss and reduces fractures during treatment	Benefit declines after discontinuation	RCTs	A	Useful in selected women; not the only osteoporosis therapy
Venous thromboembolism	Risk higher with oral estrogen; transdermal appears lower risk	Route, dose, obesity, thrombophilia and previous VTE matter	RCTs + observational	A (risk); B (route comparison)	Prefer transdermal estradiol when baseline thrombotic risk is increased
Stroke and cardiovascular events	Absolute risk varies with age and timing; MHT is not a prevention indication	Lower-dose transdermal therapy may reduce but not abolish risk	RCTs/cohorts/guidelines	A (risk); D (prevention)	Assess BP, smoking, migraine, diabetes and vascular history
Breast cancer	Risk differs by estrogen-alone vs. combined regimens and by duration	Progestogen type may modify risk; evidence largely observational	WHI follow-up/cohorts	A (risk); C (progestogen comparison)	Discuss absolute risk and reassess periodically
Endometrium and bleeding	Unopposed systemic estrogen increases endometrial risk	Adequate progestogen required with intact uterus	RCTs/guidelines	A	Investigate persistent or concerning unscheduled bleeding
Mortality and chronic disease	No formulation has a universal mortality or chronic-disease-prevention benefit	Findings cannot be generalised across ages and regimens	Long-term RCT follow-up/cohorts	D (as indication)	Do not prescribe solely to prevent dementia or cardiovascular disease

BP = Blood pressure; RCT = randomised controlled trial; VMS = vasomotor symptoms.

**Table 3:** Clinical comparison of fezolinetant and elinzanetant. FDA = US Food and Drug Administration; NK = neurokinin; VMS = vasomotor symptoms.

Feature	Fezolinetant	Elinzanetant	Interpretation
Target	Selective NK3 antagonist	Dual NK1/NK3 antagonist	Both target KNDy-related thermoregulatory signalling
Regulatory status	FDA May 2023; EU December 2023	FDA October 2025; authorised in several other jurisdictions	Local labels and reimbursement should be checked
Week-12 efficacy	About 2–3 fewer moderate-to-severe VMS episodes/day vs. placebo in pivotal trials	About 3.2 fewer episodes/day vs. placebo in OASIS 1 and 2; 1.6 fewer in OASIS 3	Cross-trial numerical differences do not permit direct efficacy ranking; no head-to-head trial exists
Sleep outcomes	Quality-of-life improvements reported; sleep is not the defining differentiator	Sleep disturbance improved in OASIS 1 and 2; long-term secondary analyses were descriptive	No head-to-head evidence establishes superiority for insomnia
Hepatic monitoring	Baseline; monthly months 1–3; months 6 and 9 in the US; boxed hepatotoxicity warning	Baseline and month 3 in the US; transaminase elevations and hepatic impairment precautions remain	Neither agent should be described as free of hepatic considerations
Other safety considerations	CYP1A2 inhibitor interactions; cirrhosis and severe renal impairment are contraindications in the US	Somnolence/daytime impairment, CYP3A4 interactions, seizure and pregnancy precautions	OASIS 4 supports VMS efficacy during endocrine therapy; it does not establish long-term oncological safety or recurrence risk
Breast-cancer endocrine therapy	Non-estrogenic; dedicated endocrine-therapy evidence is more limited	OASIS 4: week-12 placebo-adjusted difference −3.4 moderate-to-severe VMS episodes/day (95% CI −4.2 to −2.5)	Useful evidence in an oncology population; indication, interactions and local labelling still require review
Tier	A	A	Both are approved, guideline-relevant options for moderate-to-severe VMS where available

**Table 4:** Selected clinically relevant interactions in menopause pharmacotherapy. Sources are shown by row. CNS = central nervous system. The table supports structured medication review and does not substitute for current prescribing information.

Agent	Principal Mechanism/Issue	Clinically Important Combinations	Practical Action
Systemic estrogen (oral/transdermal)	Route-dependent hepatic exposure and thrombotic profile	Enzyme-inducing medicines can reduce estrogen exposure; oral therapy adds first-pass hepatic effects	Review route, interacting medicines, vascular risk, and local product information [9,13,14,15,16,25,26,27]
Fezolinetant	CYP1A2 substrate	Strong or moderate CYP1A2 inhibitors	Follow current label restrictions and hepatic monitoring [36]
Elinzanetant	CYP3A4 substrate; CNS effects	Strong CYP3A4 inhibitors/inducers; other sedating medicines	Review interactions and risk of daytime impairment [40]
Paroxetine/fluoxetine	Strong CYP2D6 inhibition	Tamoxifen (reduced endoxifen formation)	Avoid with tamoxifen; consider alternatives with less CYP2D6 inhibition [43]
Venlafaxine/citalopram/escitalopram	Lower CYP2D6 inhibition; serotonergic	Other serotonergic medicines	Often preferred with tamoxifen; monitor for serotonergic adverse effects [43]
Gabapentin	Renal elimination; sedating	Other CNS depressants; renal impairment	Dose-adjust for renal function and review additive sedation [43]
Oxybutynin	Anticholinergic burden	Other anticholinergic medicines	Review cumulative burden, particularly in older women or those with cognitive risk [44]

**Table 5:** Principal phytoestrogen classes, mechanisms, human evidence, and limitations, with clinical-actionability tiers. ERβ = estrogen receptor beta; VMS = vasomotor symptoms.

Compound/Source	Mechanistic Profile	Human Evidence	Tier	Clinical Limitations
Genistein (soy)	ERβ-preferring isoflavone; does not require conversion to equol	Selected trials report symptom changes, but recent pooled VMS effects are inconsistent or non-significant	C	Preparations, doses, outcomes and background diets vary; not an established VMS treatment
Daidzein (soy)	Parent compound that can be converted to S-equol	Mixed symptom and metabolic findings; recent pooled VMS evidence is not consistently positive	C	Response is not determined solely by equol-producing status
S-equol	Microbial metabolite with ERβ-preferring activity	Small trials/subgroup analyses suggest possible benefit in selected outcomes	C	Routine phenotype testing and clinically superior response in equol producers are unproven
Red-clover isoflavones	Mixture including formononetin and biochanin A	Meta-analyses show inconsistent or modest symptom effects	C	Extract composition and dose vary considerably
Lignans (flaxseed, sesame)	Microbial conversion to enterolactone and enterodiol	Limited trials suggest possible symptom and cardiometabolic effects	C	Dietary co-interventions and small samples limit attribution
Coumestans (legumes, alfalfa)	Estrogen-receptor activity demonstrated mainly experimentally	Insufficient clinical VMS evidence	D	Best regarded as dietary constituents rather than targeted therapy

**Table 6:** Outcome-specific interpretation of behavioural and lifestyle interventions, with clinical-actionability tiers by target domain. VMS = vasomotor symptoms; QoL = quality of life.

Intervention	VMS Frequency	VMS Bother/QoL	Other Clinically Relevant Domains	Tier	Clinical Interpretation
Menopause-specific CBT	Usually little change in objective frequency	Consistent improvement in bother and interference	Anxiety; coping; sleep	A (bother, interference)	Guideline-supported behavioural option; complements rather than mimics thermoregulatory drug effects
CBT-I	Not a VMS-frequency treatment	May improve sleep-related symptom burden	Insomnia severity and sleep quality	A (insomnia)	Target insomnia directly; evidence includes 11 RCTs in a 2025 meta-analysis
Clinical hypnosis	Some trials report reductions	Can improve severity and bother	Relaxation/coping	A (VMS)	Guideline-supported for VMS; smaller evidence base and access constraints
Exercise/resistance training	Not recommended as a VMS-specific treatment	Variable effect on symptom bother	Bone, muscle, cardiometabolic health, mood, function	A (general health); not recommended for VMS frequency	Strong general-health rationale; do not promise hot-flash reduction
Mindfulness/yoga/relaxation	Not recommended for VMS frequency	Possible subjective wellbeing benefit in selected studies	Stress, mood, flexibility, wellbeing	B (psychological outcomes); not recommended for VMS frequency	Adjunct for appropriate goals; distinguish wellbeing effects from VMS efficacy
Acupuncture	Inconsistent versus sham	May improve patient-reported symptoms in pragmatic studies	Pain and wellbeing for other indications	C	Not recommended as evidence-based VMS treatment; may be patient-preferred complementary care
Weight management	Weight loss may reduce VMS in women with overweight/obesity	May improve symptom burden	Cardiometabolic risk, sleep apnoea, mobility	A/B	Treat overweight/obesity according to established indications; not menopause-specific pharmacotherapy

**Table 7:** Clinical actionability of unsupported, emerging, and experimental strategies in menopause care that are not covered in the intervention-specific tables.

Strategy	Current Evidence	Tier	Clinical Interpretation
Routine hormonal panels in women >45 years with typical menopausal symptoms	Hormone concentrations fluctuate substantially during the menopausal transition; the 2025 European Society of Endocrinology guideline does not recommend routine biochemical testing for diagnosis or management in women >45 years with a typical clinical presentation [9].	D	Do not use routine FSH, estradiol, or AMH panels to diagnose typical perimenopause or menopause. Reserve biochemical testing for younger women, suspected early menopause/POI, or diagnostic uncertainty.
Gut microbiome sequencing/estrobolome profiling	Mechanistic and observational human evidence; the 2026 meta-analysis did not identify a consistent postmenopausal gut microbiome signature [47,48,49].	C	Research tool; not recommended for routine diagnosis or treatment selection.
Equol-producer phenotype testing	Biologically plausible microbiome-dependent metabolic phenotype, but treatment-response associations remain inconsistent [55,56].	C	Does not currently justify phenotype-guided phytoestrogen prescribing.
Targeted probiotics/synbiotics	Small heterogeneous human studies; no validated strain, dose, duration, or responder phenotype for menopause care [51,52,53].	C	May be studied experimentally but should not replace established menopause therapies.
Multi-omic/biomarker-guided treatment selection	Candidate pharmacokinetic, inflammatory, neurokinin, and microbial markers; no prospective test-guided utility trials [72].	C	Promising precision-medicine strategy, but not ready for routine care.
Faecal microbiota transplantation (FMT)	No clinical studies evaluating FMT for treatment of menopausal symptoms were identified in the literature reviewed; no menopause-specific clinical indication is established.	D	Experimental for menopause; should remain within approved indications or research protocols.
Pharmacological strategies targeting ovarian ageing	AMH/mTOR, cellular-senescence, mitochondrial, inflammatory, and follicular-activation approaches are largely preclinical or early phase [7].	D	No demonstrated ability to delay natural menopause or improve menopause-related clinical outcomes.
Targeted nano-/controlled-release hormone delivery	No clinical outcome studies evaluating nano-/controlled-release hormone delivery as a menopause treatment were identified in the literature reviewed; evidence considered here is preclinical or early translational.	D	Potential delivery concept, but efficacy, safety, and clinically meaningful tissue targeting remain experimental.

Tier C = emerging human evidence not validated for routine treatment selection; Tier D = preclinical, experimental, or currently unsupported for the stated clinical use.

## Data Availability

No new datasets were generated or analysed for this narrative review.

## Appendix A. Core Search Framework

The review was designed as a structured narrative synthesis rather than a systematic review. The original PubMed/MEDLINE search covered 1 January 2020 to 31 July 2026; targeted update searches through 20 August 2026 focused on newly published guidelines, systematic reviews, phase 3 trials, regulatory actions, and safety information. Key sources were selected hierarchically for clinical relevance: current guidelines and regulatory documents informed recommendations and approved-use/safety statements; systematic reviews and meta-analyses informed pooled evidence; pivotal randomized trials informed efficacy and safety; and landmark pre-2020 studies were identified from major guideline and review reference lists when they remained foundational to staging, mechanism, or treatment evidence. This was not a duplicate-screened systematic search, and no exhaustive denominator of screened or included studies was generated. Core search domains used for the update are shown below; exact syntax was adapted to each topic and source.

Search domain 1—core menopause care: (menopause OR perimenopause OR postmenopause) AND (guideline OR management OR treatment OR hormone therapy OR MHT).

Search domain 2—vasomotor/neurokinin therapy: (menopause AND vasomotor symptoms) AND (fezolinetant OR elinzanetant OR neurokinin OR NK3 OR NK1).

Search domain 3—microbiome/precision medicine: (menopause OR postmenopause OR genitourinary syndrome) AND (gut microbiome OR vaginal microbiome OR estrobolome OR beta-glucuronidase OR probiotic OR multi-omic OR biomarker).

Search domain 4—phytoestrogens: (menopause OR perimenopause) AND (soy OR isoflavone OR genistein OR daidzein OR equol OR phytoestrogen) AND (randomised OR systematic review OR meta-analysis).

Search domain 5—behavioural/lifestyle care: (menopause AND vasomotor OR menopause AND insomnia) AND (CBT OR CBT-I OR hypnosis OR exercise OR yoga OR mindfulness OR acupuncture OR weight loss).

Search domain 6—special populations: (menopause OR premature ovarian insufficiency OR surgical menopause) AND (breast cancer OR endocrine therapy OR BRCA OR oophorectomy OR venous thromboembolism OR thrombophilia OR obesity OR diabetes OR endometriosis).

No PRISMA flow diagram or study-count denominator is presented because study selection was not conducted as a formal systematic review. The clinical-actionability tiers used in this article are a narrative practice-readiness framework and should not be interpreted as GRADE certainty ratings.
